# Predict Postoperative Anemia of Patients: Nomogram Construction and Validation

**DOI:** 10.3389/fsurg.2022.849761

**Published:** 2022-06-09

**Authors:** Yimin Dai, Chang Han, Xisheng Weng

**Affiliations:** ^1^ Department of Orthopaedic Surgery, Peking Union Medical College Hospital, Chinese Academy of Medical Sciences & Peking Union Medical College, Beijing, China; ^2^ Peking Union Medical College, Eight-year MD program, Chinese Academy of Medical Sciences, Beijing, China

**Keywords:** anemia, predictors, nomogram, total knee arthroplasty, ESR

## Abstract

**Introduction:**

The loss of blood is a significant problem in Total Knee Arthroplasty (TKA). Anemia often occurs after such surgeries, leading to serious consequences, such as higher postoperative infection rates and longer hospital stays. Tools for predicting possible anemia can provide additional guidance in realizing better blood management of patients.

**Methods:**

2,165 patients who underwent TKA from 2015 to 2019 in the same medical center were divided into training and validation cohorts. Both univariate and multivariate logistic regression analyses were performed to identify independent preoperative risk factors for anemia. Based on these predictors, a nomogram was established using the area under the curve (AUC), calibration curve (AUC), and the area under the curve (AUC). The model was then applied to the validation cohort, and decision curve analyses (DCA) were also plotted.

**Results:**

Through analysis of both univariate and multivariate logistic regression, five independent predictors were found in the training cohort: female, relatively low BMI, low levels of preoperative hemoglobin, abnormally high levels of ESR, and simultaneously two sides of TKA in the same surgery. The AUCs of the nomogram were 74.6% (95% CI, 71.35%–77.89%) and 68.8% (95% CI, 63.37%–74.14%) of training and the validation cohorts separately. Furthermore, the calibration curves of both cohorts illustrated the consistency of the nomogram with the actual condition of anemia of patients after TKA. The DCA curve was higher for both treat-none and treat-all, further indicating the relatively high practicality of the model.

**Conclusion:**

Female, lower BMI, lower levels of preoperative Hb, simultaneous bilateral TKA, and high levels of preoperative ESR were figured out as five independent risk factors for postoperative anemia (<9.0 g/dL) in patients undergoing TKA. Based on the findings, a practical nomogram was constructed to predict risk of postoperative anemia. The evidence level should be level 4 according to guideline.

## Introduction

Total knee arthroplasty (TKA) is widely performed to improve mobility and quality of life for symptomatic knee osteoarthritis patients. More than 500,000 knee replacements are performed annually in the USA (1). This number was projected to grow by 673% from 2005 to 2030 (2). Patients undergoing TKA are often elderly, suffer from comorbidities and poorer recovery ability. A significant proportion of these patients have preoperative anemia and the trauma caused by surgery is great. All these factors tend to cause or aggravate postoperative anemia. Postoperative anemia in itself is considered an independent risk factor predisposing to poor prognosis, including higher postoperative infection rates (3), longer hospital stays (4), higher mortality rates (5), longer durations of rehabilitation (4) and worse quality of life (6).

A significant number of frail older people with complex co-morbid conditions are currently receiving knee arthroplasty. High-risk patients must be recognized early so that measures may be implemented to reduce the risk of anemia. The treatment involves the treatment of underlying diseases, a balanced diet and the use of erythropoietin and iron. However, there is still no simple and easy method for evaluating the risk of postoperative anemia in clinical practice. Whether to use erythropoietin and iron for outpatients continues to perplex orthopedists.

We aimed to investigate the incidence and preoperative risk factors for postoperative anemia in patients following TKA and develop a cost-efficient dynamic nomogram to be implemented into clinical practice.

## Materials and Methods

From June 2016 to June 2019, 2,166 patients who received TKA in the same medical center were retrospectively reviewed. The inclusion criteria included: (1) diagnosed with osteoarthritis independently by two orthopedic doctors that met surgery indications; (2) received primary TKA that was performed by the same team; (3) accurate records of previous medical history; (4) received no extra iron supplements or blood transfusions prior to admission. All patients were given the same intraoperative blood management measures, such as tranexamic acid administration. Patients who met one of the following criteria were excluded: (1) revision arthroplasty; (2) coagulation disorders; (3) patients with preoperative Hb < 9.0 g/dL. This study was approved by the Ethics Committee of Peking Union Medical College Hospital. All patients signed informed consent forms before surgery.

### Surgical Procedure

After being diagnosed with osteoarthritis in our hospital, patients who met the surgery indication would be given a booklet on perioperative information of knee surgery to make sure them were fully informed of their own illness. Once patients chose to receive TKA, preoperative evaluation would be performed about two weeks before surgery, including a necessary imaging examination, bone density test, serum hemoglobin (Hb), erythrocyte sedimentation rate (ESR), etc. Close monitoring will be required if Hb < 9.0 g/dL, and 1. ferralia and erythropoietin (EPO) 2. transfusion would be given if necessary.

All patients enrolled in this study underwent standard TKA performed in the supine position by two orthopedists from the same hospital with over 30-year experience. Tourniquets were commonly used during the operations, and the pressure was set to 60 mmHg. Posterior cruciate stabilizing (PS) prosthesis was used in the operation. Physical exercise and continuous passive motion exercise device (CPM) were applied on post-operative day 1 until discharge, which were performed twice daily according to standard guidelines (7).

### Study Variable

We used a total of 24 variables for the modeling analysis in this study. Postoperative anemia is defined as Postoperative Hb < 9.0 g/dLl. The patient-related characteristics included sex, age, body mass index (BMI), smoking status and comorbidities. Smoking was defined as the number of times a patient reported smoking cigarettes in the year before admission for TKA. Comorbidities may have an important impact on survival, and comorbidity scores are often implemented in studies assessing prognosis.

This study used the Charlson-based ICD-10 co-morbidity instrument developed by the Royal College of Surgeons of England (8). It reflects current understanding of the prognostic impact of co-morbid disease and aims explicitly to avoid misclassifying surgical complications as co-morbidities. The comorbidities involved Myocardial infarction, Congestive cardiac failure, Peripheral vascular disease, Cerebrovascular disease, Dementia, Chronic pulmonary disease, Rheumatological disease, Liver disease, Diabetes mellitus, Hemiplegia or paraplegia, Renal disease, Any malignancy, Metastatic solid tumor and AIDS/HIV. All available information was collected before surgery.

### Statistical Analysis

Patients were randomly divided into a training cohort and a validation cohort with a ratio of 7:3. Using the training cohort, the logistic regression models were constructed by incorporating these variables. Performance of the models in both the training cohort and validation cohort included discrimination and calibration. Discrimination is the predictive accuracy of distinguishing patients with anemia from those without anemia and can be measured by the area under the receiver operating characteristic (ROC) curve (AUC).

Calibration curves were plotted using 1,000 bootstrap resamples, which reflected the model’s agreement between the predicted probability and the actual probability. Decision curve analysis (DCA) was used to assess the model’s clinical usefulness. Based on the logistic regression model, a dynamic nomogram was developed, presenting a specific mechanism for calculating the risk of postoperative anemia.

Continuous data were expressed as median and interquartile (IQR). Statistical analysis was performed using IBM SPSS 25.0 (SPSS Inc; Chicago, IL, USA) and R software version 3.6.3. Pearson’s Chi-square test was used for categorical data analysis (Fisher exact test was used if necessary), while Mann–Whitney U-test was used for comparing quantitative parameters. *P* < 0.05 were considered statistically significant. All statistical analyses were two-sided.

## Results

### Basic Variables and Information of Patients

A total of 3,547 consecutive patients who underwent TKA at Peking Union Medical College from June 2015 to June 2019 were retrospected, while 2,165 of them met the inclusion criteria; in chronological order, 1,517 were included in the training group and the rest were in the validation group. The ages of patients varied between 20 and 94, while the mean age was 66.9; they were divided into five subgroups: younger than 60 years old, 60–65, 65–70, 70–75, and over 75 years old; while the patients aged 65–70 were the most, up to 27.8%. There were 1,795 females and 370 males. 159 and 79 patients had the habit of smoking and drinking separately, and 260 patients took drugs regularly. The average BMI was 27.01 kg/m^2^, varied between 14.7–42.9 kg/m^2^. The proportions of different constitutions from both cohorts were quite similar. Detailed information about the variables and patients is displayed in Table 1.

**Table 1:** Basic characteristics of patients from both training and validation cohort.

Variables	Training cohort		Validation cohort		*p-*value
	*N*	%	*N*	%	
	**1,517**	**70.1**	**648**	**29.9**	
Age (years)					0.948
<60	206	(13.6)	85	(13.1)	
60–65	293	(19.3)	135	(20.8)	
65–70	422	(27.8)	180	(27.8)	
70–75	302	(19.9)	125	(19.3)	
>75	294	(19.4)	123	(19.0)	
Sex					0.366
Male	1,395	(92.0)	611	(94.3)	
Female	122	(8.0)	37	(5.7)	
BMI (kg/m^2^)					0.136
<24	280	(18.5)	143	(22.1)	
24–28	655	(43.0)	273	(42.1)	
>28	584	(35.8)	232	(35.8)	
Smoking					0.069
Yes	122	(8.0)	37	(5.7)	
No	1,395	(92.0)	611	(94.3)	
Drinking					0.591
Yes	58	(3.8)	21	(3.2)	
No	1,459	(96.2)	627	(96.8)	
Drug					0.335
Yes	175	(11.5)	85	(13.1)	
No	1,342	(88.5)	563	(86.9)	
Infusion of protein					0.679
Yes	408	(26.9)	168	(25.9)	
No	1,109	(73.1)	480	(74.1)	
Abnormally high CRP levels					0.912
Yes	197	(13)	86	(13.3)	
No	1,320	(87)	562	(86.7)	
Abnormally high ESR levels					0.451
Yes	357	(23.5)	163	(25.2)	
No	1,160	(76.5)	485	(74.8)	
Hb < 12.0 g/dL (male) or <11.0 g/dL (female) (preoperative anemia or not)					0.861
Yes	66	(4.4)	30	(4.6)	
No	1,451	(95.6)	618	(95.4)	
Sides of TKA					0.058
One side	494	(32.6)	239	(36.9)	
Two sides	1,023	(67.4)	409	(63.1)	
Number of comorbidities					0.97
<2 kinds of comorbidities	732	(48.3)	313	(48.3)	
2 kinds of comorbidities	385	(25.4)	167	(25.8)	
>2 kinds of comorbidities	400	(26.4)	168	(25.9)	
Anemia in three days after operation					0.778
Yes	253	(16.7)	112	(17.3)	
No	1,264	(83.3)	536	(82.7)	

*Data are n (%) or mean ± SD. Bold represents p-value of the variable is less than 0.05*.

### Possible Risk Factors for Postoperative Anemia

Anemia occurred in 253 patients in the training cohort within 3 days after TKA, accounting for 16.7% of the cohort. Several potential risk factors were identified for postoperative anemia by regression analyses. In the training cohort, sex, Body Mass Index (BMI), Erythrocyte Sedimentation Rate (ESR), Hemoglobin (Hb) and the sides of TKA were found to be associated with postoperative anemia by univariate logistic regression analysis. These variables were further analyzed by multivariable logistic regression; independent risk factors were ultimately identified as sex, BMI, hemoglobin and sides of TKA (Table 2). For sex of patients, females were more likely to suffer from anemia after operation (adjusted odds ratio: 0.458, 95% CI, 0.289–0.727, *p* < 0.001). BMI was another predictor; patients with lower BMI tended to have higher rates of postoperative anemia, considering the contrast between BMI < 24 kg/m^2^ and BMI > 28 kg/m^2^ (adjusted OR: 0.312, 95% CI, 0.206–0.473, *p* < 0.001). The ones with 24 < BMI < 28 kg/m^2^ also had lower rates of postoperative anemia, though the results were not quite significant (*p* > 0.01). In addition, lower levels of preoperative Hb and both sides rather than one side of TKA suggested possible postoperative anemia separately (former adjusted OR: 5.671, 95% CI, 3.255, *p* < 0.001; latter adjusted OR: 4.247, 95% CI, 3.156–5.714, *p* < 0.001). Surprisingly, the influence of comorbidities was not as significant as people commonly think; patients with over one to two comorbidities didn’t show higher rates of anemia, and though those with more than three comorbidities did suffer from anemia more easily, the results weren’t significant enough (OR: 1.162, 95% CI, 0.842–1.602, *p* = 0.361).

**Table 2:** Postoperative anemia of TKA: univariate and multivariate analysis of possible risk factors in the training cohort.

Variables	Univariate analysis	*p-*value	Multivariate analysis	*p-*value
	OR (95% CI)		Adjusted OR (95% CI)	
Age (years)				
<60	1.000			
60–65	1.131 (0.709–1.804)	0.605		
65–70	0.955 (0.617–1.480)	0.838		
70–75	0.977 (0.655–1.459)	0.911		
>75	0.992 (0.644–1.527)	0.971		
Sex				
Female	1.000		1.000	
Male	0.440 (0.283–0.686)	**<0.001**	0.458 (0.289–0.727)	**<0.001**
Drug				
No	1.000			
Yes	0.732 (0.461–1.160)	0.732		
BMI (kg/m^2^)				
<24	1.000		1.000	
24–28	0.832 (0.591–1.169)	0.289	0.683 (0.471–0.991)	**0.045**
>28	0.417 (0.284–0.611)	**0**	0.312 (0.206–0.473)	**<0.001**
Infusion of protein				
No	1			
Yes	0.793 (0.590–1.065)	0.123		
Abnormally high CRP levels				
No	1			
Yes	0.880 (0.596–1.299)	0.520		
Hb < 12.0 g/dL (male) or <11.0 g/dL (female) (preoperative anemia or not)				
No	1			
Yes	4.903 (2.962–8.117)	**<0.001**	5.671 (3.156–5.714)	
Abnormally high ESR levels				
No	1.000		1.000	
Yes	1.537 (1.140–2.073)	**0.005**		
Sides of TKA				
One side	1			
Two sides	3.740 (2.829–4.943)	**<0.001**	4.247 (3.156–5.714)	**<0.001**
Number of comorbidities				
<2 kinds of comorbidities	1.000			
2 kinds of comorbidities	0.999 (0.714–1.397)	0.994		
>2 kinds of comorbidities	1.162 (0.942–1.602)	0.361		
Anemia in three days after operation				
No	1,264 (83.3%)			
Yes	253 (16.7%)			

A similar analysis was performed in the validation group, as shown in Table 3. Sex, preoperative Hb levels and sides of TKA were still strong risk factors. However, BMI did not show comparable effects, while ESR did; in univariate analysis, high ESR was strongly associated with worse ending of anemia, though such associations slightly weakened in multivariable logistic regression (OR: 1.666, 95% CI, 1.043–2.66, *p* = 0.033).

**Table 3:** Postoperative anemia of TKA: univariate and multivariate analysis of possible risk factors in the validation cohort.

Variables	Univariate analysis	*p-*value	Multivariate analysis	*p-*value
	OR (95% CI)		Adjusted OR (95% CI)	
Age (years)				
<60	1.000			
60–65	0.764 (0.385–1.520)	0.443		
65–70	0.836 (0.439–1.589)	0.584		
70–75	0.709 (0.350–1.437)	0.340		
>75	0.638 (0.310–1.313)	0.222		
Sex				
Female	1.000		1.000	
Male	0.306 (0.138–0.678)	**0.004**	0.399 (0.177–0.900)	**0.027**
Drug				
No	1.000			
Yes	0.846 (0.451–1.588)	0.603		
BMI (kg/m^2^)				
<24	1.000			
24–28	0.747 (0.440–1.266)	0.278		
>28	0.908 (0.534–1.544)	0.721		
Infusion of protein				
No	1.000			
Yes	0.697 (0.424–1.145)	0.154		
Abnormally high CRP levels				
No	1.000			
Yes	1.211 (0.682–2.152)	0.514		
Hb < 12.0 g/dL (male) or <11.0 g/dL (female) (preoperative anemia or not)				
No	1.000		1.000	
Yes	6.214 (2.937–13.15)	**<0.001**	6.152 (2.724–13.89)	**<0.001**
Abnormally high ESR levels				
No	1.000	1.000		
Yes	2.160 (1.403–3.326)	**0.000**	1.666 (1.043–2.66)	0.033
Sides of TKA				
One side	1.000			
Two sides	2.63 (1.737–3.981)	**<0.001**	2.90 (1.866–4.495)	**<0.001**
Number of comorbidities				
<2 kinds of comorbidities	1.000			
2 kinds of comorbidities	0.946 (0.570–1.571)	0.830		
>2 kinds of comorbidities	1.154 (0.710–1.875)	0.562		

### Establishment and Validation of the Nomogram to Predict Anemia Ending

**Figure 1:**
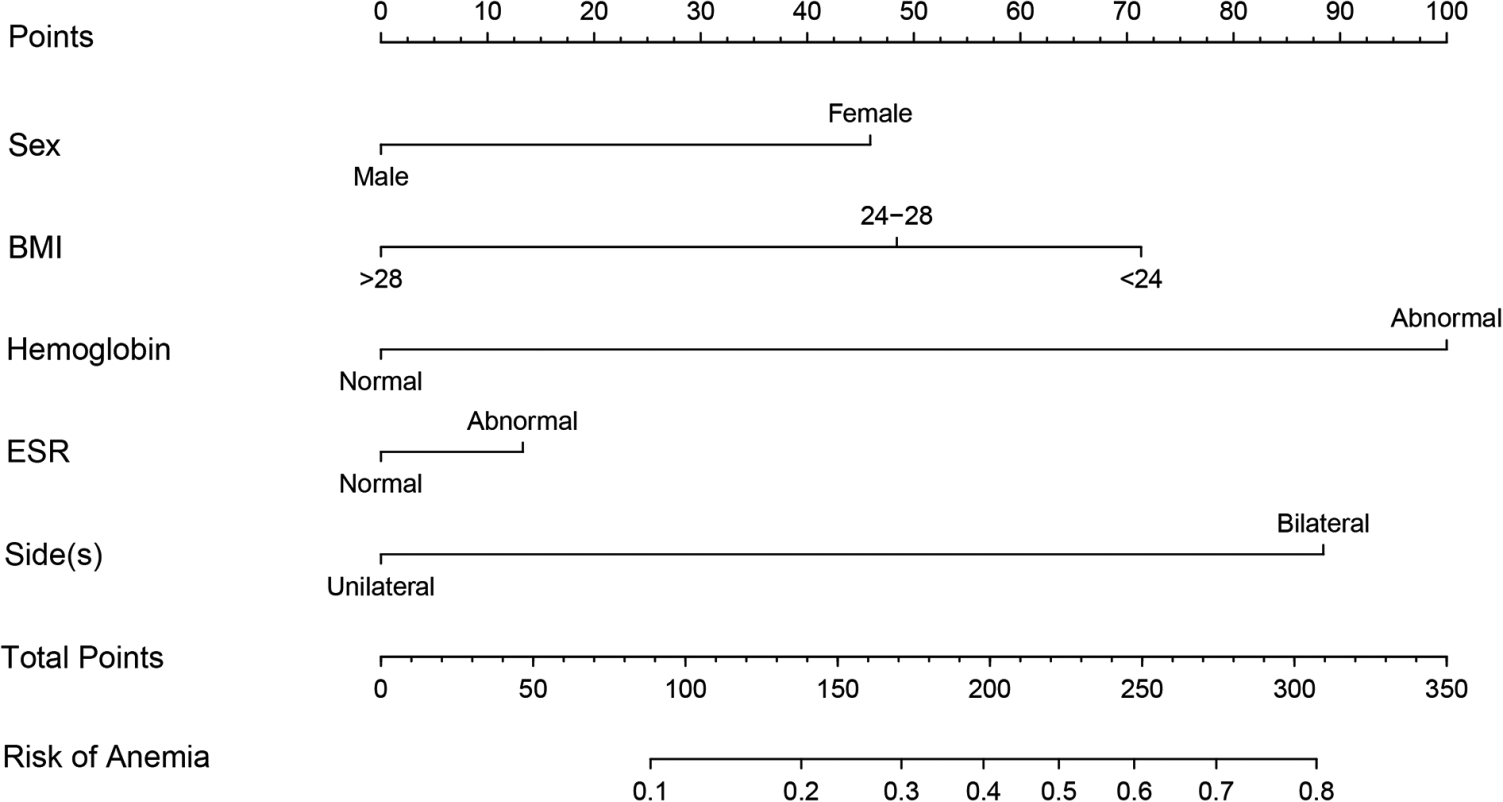
The nomogram for calculating risk of postoperative anemia of patients who underwent TKA.

Based on the final results of multivariate analysis, the nomogram was established and displayed in Figure 1. The total point of every given patient could be calculated from the nomogram, which was the sum of each risk factor mentioned above. Possibility of postoperative anemia was predicted by the final score.

**Figure 2:**
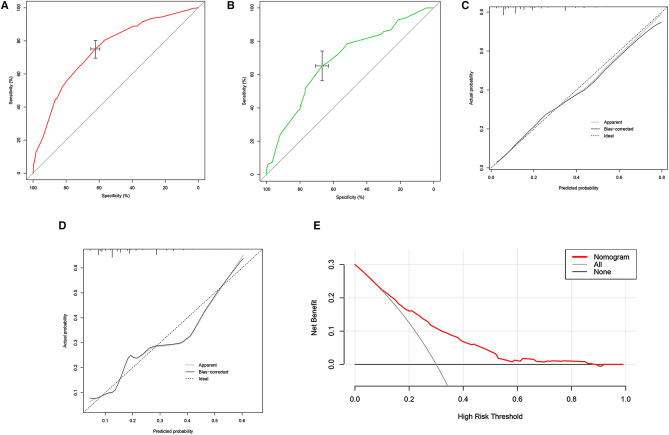
The receiver operating characteristic (ROC) curve of training cohort (**A**) validation cohort (**B**), the calibration curve of training cohort (**C**) and validation cohort (**B**), and decision curve analysis (DCA) of training set (**E**).

To ensure the feasibility and accuracy of the nomogram, we performed discrimination and calibration assessments for the model. For discrimination, the ROC curves of the nomogram applied to both training and validation cohorts were plotted to perform validation internally and externally, together with the AUC values (Figures 2A,B), which showed relatively high predictive accuracy (internal validation, AUC: 74.6%, 95% CI, 71.35%–77.89%; external validation, AUC: 68.8%, 95% CI, 63.37%–74.14%). Moreover, the calibration of both internal and external curves also demonstrated high consistency between the monogram and practicalities (Figures 2C,D). Though with a high AUC value, we further performed Decision Curve Analysis (DCA), which indicated that the nomogram did predict anemia outcome better than no model for predicting (Figure 2E).

## Discussion

A nomogram was established using five preoperative factors to predict postoperative anemia in patients who underwent TKA. Before and after TKA, it would probably be a useful tool for better blood management of individualized patients.

This study was based on previous predictive models with some improvements. First of all, at the level of method design, the endpoint was widened from transfusion to anemia, which differed from previous researches (9–13). Those who had postoperative Hb lower than 90 g/L but didn’t meet the indications for transfusion were not usually included in previous researches, yet their relatively mild anemia had also been identified to adversely influence the recovery, discharge and life quality change after orthopedic surgeries (14); also, they could do damage to major organs such as kidney, heart, brain, etc. (15). Indeed, even no surgeries were performed, anemia that did not need transfusion was found to be associated with increased risk of hospitalization and mortality of any cause; and anemic elderly people tended to be in worse condition for cognition and mood (16). All in all, previous studies had strongly suggested long-term side effects of anemia on patients who did not need transfusion. Thus, it is worth for surgeons to know that no need for transfusion does not mean no demand for blood management; and undoubtedly, this study provided a better predictive method for blood management of patients, including consideration of side effects of anemia which did not need transfusion. In contrast to models which simply included preoperative hemoglobin as the cut-off value for transfusion (17), the nomogram in this study consisting of multiple predictors is even more advantageous in comprehensively evaluating patients’ conditions. In terms of participants, the nearly three thousand ones ensured the accuracy and feasibility of the nomogram to a certain extent; and investigating both unilateral and bilateral TKA provided more reasonable evidence in explaining postoperative anemia than simply covering unilateral ones (9, 11, 13). In addition, we ensured that patients in both the training and validation cohorts received the operation in relatively the same period of time, thus preventing interference of changing with the operation and ensuring the stability of baseline.

After both univariate and multivariate regression analyses, a total of five risk factors were found to be associated with postoperative anemia: female sex, low BMI, low preoperative hemoglobin level, high levels of ESR, and simultaneous bilateral TKA. These predictors were in line with our perceived common sense, and were also mentioned in several previous studies. Females tended to have lower Hb levels before surgery; Steuber et al. identified females accounting for 92% of patients with pre-operative iron deficiency anemia (18). Biboulet et.al found blood transfusion was more common in females through univariate analysis, though such significance disappeared in multivariate analysis (19). Another group of researchers obtained similar results that female patients were more likely to receive transfusion than males (20); thus, it’s reasonable that we included sex as one of the predictors in our nomogram.

Low BMI quantifies underweight as reflecting malnutrition to a certain extent. There have already been multiple studies showing people with lower BMI were at higher risk of anemia, while in contrast, overweight and obesity were inversely correlated with anemia (21–23). In research specifically accessing in-hospital outcomes among underweight patients after operations, low BMI was particularly pointed out to be an important risk factor for poor clinical outcomes including postoperative anemia, considering that such patients were more prone to poorer nutritional reserves and more susceptible to other comorbidities (24). However, not all similar predictive models considered low BMI as a significant risk factor (9, 20); and those models covering BMI didn’t agree on the same cut-off value with each other (11, 25, 26), neither of us. This was probably resulted from the differences among participants. In our study, we separated patients into 3 groups of different BMI ranges and identified their contributions to postoperative anemia, which were feasible in both training and validation cohorts.

Among the five, preoperative hemoglobin seems to be the most influential predictor; this has already been validated in multiple previous studies. Gu et.al revealed that preoperative mild anemia was significantly associated with a series of postoperative complications including anemia which required transfusion (27). Similar conclusions were also given by other group of researchers (13, 28), and predictive models included the factor as key predictor (10, 29). Specifically, in our research, we excluded those patients with moderate to severe anemia to prevent possible complex interferences, and set the observational endpoint as postoperative anemia rather than transfusion; even so, mild anemia before operation still had powerful influence. It’s easy to understand that anemic patients themselves should have disorders in the production of hemoglobin; and TKA, a blood-consuming operation undoubtedly could not correct such disorders but promote their exacerbation.

A relatively surprising finding turned out to be the effect of ESR; few studies and predictive models mentioned it. In our nomogram, this factor did not show as strong an effect as other factors, yet it remained significant after both univariate as well as multivariate regression analyses. Cathrin et.al pointed out that anemia frequently accompanied chronic diseases, which were related to systemic inflammation, as reflected by markers like ESR (30). Examples of possible chronic diseases include chronic obstructive pulmonary diseases (COPD), and anemia was found to be independently correlated with ESR in COPD patients (31). In our study, patients undergoing TKA were relatively elderly and had a relatively high probability of suffering from chronic diseases.

Finally, bilateral TKA in the same operation was a significant risk factor compared to unilateral one, and the effect was only secondary to preoperative anemia. Relevant researches were plenty, and above-mentioned effect could be understood easily. Many models contained sides of TKA (10, 25, 29); in bilateral TKA, time for operation tended to be longer, and more autologous blood could be lost, which could probably explain such effects.

Since the predictive model was constructed, indicating the clinical utility of the model seems to be quite significant, though high AUC values were received; thus, DCA was performed, showing that the nomogram presented a net benefit which was much higher than both the treat-none and treat-all lines. This is significant for developing countries, considering the possible economic burden from ferralia consumption. Therefore, the nomogram could be a utilitarian tool for clinicians to better perform blood management in advanced and timely care to prevent severe endings of anemia.

Several factors were identified to be irrelevant for postoperative anemia, such as age, smoking, drinking, drug taking, high CRP levels, comorbidities, etc. In previous researches, predictors like age, comorbidities, etc showed significant results and were included in the predictive models in previous researches (10, 11, 32); unfortunately, such relevance was not identified in this study, along with other factors such as high CRP levels, smoking, drug taking. The varied results across studies could be explained by diverse composition of patients, different tyoes of surgeries as well as the varied design of the studies. Particularly, postoperative anemia was not found to be associated with comorbidities in this study. According to previous studies, diabetes, one of the classic chronic comorbidities, was identified to be related to anemia through functional erythropoietin deficiency (33); others also identified possible interactions between coronary heart diseases and anemia (34). However, those with serious comorbidities were not able to receive the operation and were excluded from the very beginning. As a selective operation, TKA was performed to improve the life quality of patients who would otherwise not have received the operation, and to improve management of comorbidities. Thus, it is reasonable to believe that comorbidities may not be associated with postoperative anemia in such well-managed patients.

There are some limitations in this study. First of all, this is a single-center study without external validation in other medical centers. This weakened the feasibility of our model to a certain extent; we therefore welcome colleagues from all over the world to try the nomogram in perioperative blood management for patients undergoing TKA. Secondly, though more than 2,000 patients were included in the study, the sample size was still limited. More patients are expected to be studied to generate even better results in the future.

## Conclusion

In this study, female, lower BMI, lower preoperative Hb levels, simultaneous bilateral TKA, and higher levels of preoperative ESR were identified as five independent risk factors for postoperative anemia (<9.0 g/dL) in patients undergoing TKA. This is a typical clinical study of level 4 of evidence. To better predict risk of such postoperative anemia, a nomogram integrating all of the factors was constructed which could greatly help to predict the risk of postoperative anemia requiring intervention.

## Data Availability

The original contributions presented in the study are included in the article/Supplementary Material, further inquiries can be directed to the corresponding author/s.
